# Usefulness of Acceptability and Feasibility Assessment in Studies of Nursing Interventions*

**DOI:** 10.17533/udea.iee.v41n1e02

**Published:** 2023-03-14

**Authors:** Claudia Milena Garizábalo-Dávila, Alba Luz Rodríguez-Acelas, Wilson Cañon-Montañez

**Affiliations:** 1 RN, M.Sc., Ph.D. Assistant Professor, Department of Health Sciences, Universidad de la Costa, Barranquilla, Colombia. Email: cgarizab2@cuc.edu.co. Corporación Universitaria de la Costa Department of Health Sciences Universidad de la Costa Barranquilla Colombia cgarizab2@cuc.edu.co; 2 RN, M.Sc., Ph.D. Associate Professor. Faculty of Nursing, Universidad de Antioquia, Medellín, Colombia Email: aluz.rodriguez@udea.edu.co Universidad de Antioquia Faculty of Nursing Universidad de Antioquia Medellín Colombia aluz.rodriguez@udea.edu.co; 3 RN, M.Sc., Ph.D. Associate Professor. Faculty of Nursing, Universidad de Antioquia, Medellín, Colombia Email: wilson.canon@udea.edu.co Universidad de Antioquia Faculty of Nursing Universidad de Antioquia Medellín Colombia wilson.canon@udea.edu.co

Nursing interventions constitute an essential component in the discipline and play a central role in the distinction of the nursing practice with regards to other health professionals. In light of the theory, authors like Burns and Grove,^1^ define nursing interventions as deliberate cognitive, physical or verbal activities, which are implemented in individuals and families, seeking therapeutic objectives that contribute to health and wellbeing. In turn, for Sidani and Braden,^2^ these are described as treatments, therapies, procedures, or actions developed by health professionals within a specific situation of the patient, for the purpose of modifying current conditions and leading to beneficial health outcomes. These approaches aim, in addition to guiding professional to respond to the needs of individuals and/or families, to move towards evaluating the achievement of the results; for this, structured, systematic and rigorous evaluation processes of the interventions are required. 

In this sense, Campbell *et al.,*^3^ designed a framework for the design and evaluation of complex interventions to improve health, which contemplate two key elements for the systematic evaluation of the interventions; immersed in Phase-II studies; these are: i) the feasibility of delivering the intervention and ii) acceptability by providers and patients regarding the intervention, in order to achieve optimal efficacy. Similarly, Sidani and Braden^2^ report that the evaluation of interventions requires a feasibility and acceptability phase of the intervention to assess the efficacy or effectiveness of achieving expected results. 

Feasibility refers to the practicality of administering the treatment; for example, in an intervention of social support to the elderly, which aims to improve self-management of type-2 diabetes mellitus,^4^ its components and activities are implemented satisfactorily, bearing in mind the dose (number and interval of educational sessions) and suggested manner of administration (individualized delivery strategies, face to face, use of educational material). Acceptability is the perception of patients and health professionals versus determining whether the intervention is appropriate to address the problem, in reasonable, adequate, and convenient manner for its application in daily life; hence, these two aspects are used and tested in practice - initially, in pilot studies, seeking to evaluate the response of acceptability and feasibility of nursing interventions. 

According to these authors, the operationalization of the acceptability of nursing interventions occurs through two paths, one inductive and the other deductive. The inductive path looks for the participation of the main actors of the intervention under study (patients and health professionals responsible for care) in a space for dialogue and discussion on the central elements of the intervention: type of interventionist, time, scenario, delivery mode, dose, objectives, components, and activities to achieve the goals in this dialogue setting, using open and closed questions as an opinion strategy of the actors. For example, the study by Whittemore *et al*.,^5^ conducted the mHealth intervention; in said research, acceptability was evaluated through interviews and focal groups with the actors of the intervention; that is, expert health professionals and patients, which determined the challenges to manage diabetes mellitus and the needs of the intervention’s content. Among the recommendations suggested by adults with diabetes and health professionals, these focused on the need for more information about diabetes, pharmacological and non-pharmacological treatment, ways to prevent complications and the need for social support. Likewise, health professionals suggested facilitating the comprehension of the content of the intervention through visual messages, increasing the repetition of the information, having a pleasant environment when providing the sessions and - finally - the inclusion of family support.^5^

The second path to opt for the intervention’s acceptability is through a deductive focus, where the principal actors contribute by giving quantitative value to their appreciations regarding the suitability, relevance, and usefulness of the intervention. The pilot study by Cossette *et al.,*^6^ for the acceptability of an intervention in patients with heart failure and their caregivers, used the Treatment Acceptability and Preference Questionnaire, which evaluated if the intervention was appropriate, acceptable, and effective through responses in a 5-point Likert scale. 

Regarding the feasibility of the interventions, a key element in the assessment of nursing interventions, it is operationalized by monitoring compliance with each of the elements of the intervention; includes aspects, like making sure the interventionists are trained to perform their role. It begins with an intervention protocol that guides the intervention plan, which must be clear, organized and specific about each session of the intervention; besides, it is necessary to train the interventionists on the conceptualization and operationalization of the intervention. Other aspects to keep in mind for the feasibility of the interventions are the material resources, which can be electronic devices (computer equipment, projectors, etc.); printed material, and general supplies (medical and non-medical); similarly, the physical and social environment in which the intervention takes place gains importance; among these, there are the location of the place to carry out the intervention, the internal adaptations of the place (elevator, furniture, context). 

The last aspect for the feasibility of the interventions is fidelity; that is, that the intervention is delivered as designed. For fidelity of the intervention, Sidani and Braden^2^ propose two methods to monitor it: theoretical fidelity and operational fidelity. The theoretical method consists in articulating the active ingredients, components, activities, and actions of the intervention; according with the theory that supports the intervention, it is operationalized through two strategies: i) construction of a matrix that contains the elements of the intervention described and ii) validation of the content of the intervention, using the matrix as input. The operational fidelity examines the performance of the interventionists and the patients’ adherence to the practices developed.^2^ In this regard, the Treatment Fidelity Workgroup of the National Institutes of Health (NIH) Behavior Change Consortium recommends for this type of fidelity to examine the delivery, reception, and compliance of the intervention.^7^ An example of such is the protocol of a randomized controlled trial for a social support intervention for self-management of type-2 diabetes mellitus.^4^

In synthesis, each of the methodologies proposed to evaluate nursing interventions reviewed in this editorial article, become relevant inasmuch as they seek to provide support based scientific evidence, aiming to contribute significantly in addressing the needs of individuals, families, caregivers, groups, and populations. Likewise, the generation of efficient and effective interventions is a valuable resource for nursing professionals to have greater tools in the qualification of care in the different contexts of practice, leading to progress in the quality of care and visibility of the work of the scientific discipline of nursing.
